# Establishing an experimental model approach to thermal-induced spinal cord injury in mice

**DOI:** 10.3389/fncel.2026.1779728

**Published:** 2026-03-17

**Authors:** Arata Mashima, Kazuya Yokota, Kazu Kobayakawa, Hirokazu Saiwai, Kazuki Kitade, Jun Kishikawa, Mami Sugano, Shintaro Sasaguri, Kiyoshi Tarukado, Kenichi Kawaguchi, Gentaro Ono, Takeshi Maeda, Yasuharu Nakashima

**Affiliations:** 1 Department of Orthopaedic Surgery, Graduate School of Medical Sciences, Kyushu University, Fukuoka, Japan; 2 Department of Orthopaedic Surgery, Japan Organization of Occupational Health and Safety Spinal Injuries Center, Iizuka, Japan

**Keywords:** animal model, collagen, fibrotic scar, spinal cord injury, thermal injury

## Abstract

Neurological deficits following spinal surgery represent a severe complication, and thermal damage from high-speed drills is considered a potential cause, but the underlying pathophysiology remains poorly understood. Here, we aimed to develop and characterize a novel mouse model of thermal-induced spinal cord injury (TiSCI). Given that surgical drilling can generate temperatures of 90 °C, we created a TiSCI model by applying a controlled thermal exposure (90 °C for 1 min) to the exposed thoracic cord in mice. The TiSCI model induced significant and persistent hindlimb motor deficits, accompanied by marked demyelination and progressive collagen deposition at the lesion site. Transcriptomic analysis by RNA-sequencing revealed that this pathology was associated with a significant upregulation of pro-fibrotic genes, including Col1a1, Col1a2, Tgfβ1, and Acta2. Using Col1a2-EGFP transgenic mice, we identified a prominent fibrotic scar composed of Type I collagen-producing cells at the lesion site, evident by 7 and 14 days post-injury, which spatially overlapped with demyelinated regions devoid of axons. KEGG pathway analysis highlighted pathways related to extracellular matrix organization, phagocytosis, and fibroblast activation. Notably, Scarb3 and Actg2 were upregulated early, while Itgax and Fzd7 were induced later, implicating both immune cell responses and Wnt/β-catenin signaling in fibrotic scar progression. In conclusion, this study established an experimental platform for investigating TiSCI in mice, providing first direct evidence that a thermal insult causes persistent neurological deficits by inducing a robust fibrotic response. The resulting collagenous scar acts as a physical barrier to axonal connectivity, establishing the fibrotic process as a key therapeutic target.

## Highlights

- We established a novel experimental model approach for thermal-induced spinal cord injury (TiSCI).
- TiSCI triggers a pro-fibrotic gene response, forming a dense Type I collagen scar.
- The fibrotic scar disrupts axonal tracts, causing functional deficits after TiSCI.
- Transcriptomics reveals key pathways driving robust fibrotic response after TiSCI.

## Introduction

Surgical intervention, including decompression and fusion, is a common treatment for spinal disorders resulting from degenerative changes and trauma. Currently, over one million spinal surgeries are performed annually in the United States (Dykhouse et al., 2024). Although spinal surgery aims to restore neurological function and spinal column stability, some patients unfortunately develop postoperative neurological deficits resulting from spinal cord injury (SCI), even in the absence of any apparent direct mechanical injury to neural elements (Zhang et al., 2013; Liao et al., 2020). Due to the limited regenerative potential of the central nervous system, SCI often results in permanent neurological deficits, imposing a substantial burden on patients (Ahuja et al., 2017). Despite its clinical significance, the fundamental causes and molecular mechanisms underlying postoperative paralysis following spinal surgery remain unclear.

High-speed drills are essential and widely-used instruments in modern spinal surgery (Pait et al., 1991; Walker et al., 2019). However, drilling generates substantial heat at the interface between the drill tip and bone tissue (Kondo et al., 2000; Tamai et al., 2017). This thermal exposure can cause injury to neural elements, leading to cell death and tissue damage. Previous studies report postoperative paralysis occurs in approximately 1 to 2% of all spinal surgeries (Cramer et al., 2009; Takeuchi et al., 2025). Although direct surgical trauma to the neural elements and compressive hematoma have been reported as potential causes, drill-induced thermal spinal cord injury is a suggested complication in clinical practice (Hosono et al., 2009; Yamamoto et al., 2013; Takahashi et al., 2025); yet, supporting evidence from clinical data remains scarce and inconclusive. This clinical uncertainty highlights the urgent need for a controlled animal model to definitively test the hypothesis that heat generated during spinal drilling can cause SCI and to elucidate its underlying mechanisms.

Despite this clinical concern, no reproducible model has yet been established that accurately represents SCI caused by controlled thermal exposure. To address this gap, we developed a novel experimental model approach in which quantifiable thermal energy is applied directly to the spinal cord in a reproducible manner. In this study, we describe the establishment and comprehensive characterization of thermal-induced spinal cord injury (TiSCI) model. Furthermore, we present an initial investigation into the resulting pathology to explore potential therapeutic targets.

## Materials and methods

### Mice

Wild-type C57/BL6N mice were purchased from Japan SLC (Shizuoka, Japan). Col1a2-EGFP transgenic mice (C57BL/6 background), generated and kindly donated by Dr. Yutaka Inagaki (Higashiyama et al., 2009), were also used. All experiments used female mice aged 8 to 10 weeks. A total of 132 age-matched mice were randomly assigned to the spinal cord injury (SCI) group. All mice were housed under controlled conditions of humidity (30–70%), temperature (21–23 °C), and a 12-h light/dark cycle. All experimental procedures were conducted in compliance with animal protocols approved by the Committee of Ethics on Animal Experiments of the Faculty of Medicine, Kyushu University (A24-398-2). Standard exclusion criteria were established for animals exhibiting severe postoperative complications, such as extensive autophagia, infection, or excessive weight loss; however, no animals met these criteria in the present study. All measurements were performed by an experimenter blinded to the group assignments.

### Measurement of high-speed drill temperature during bone drilling

To measure the temperature of the high-speed drill, a neurosurgical power system (ELAN 4 electro, AESCULAP) was set to 65,000 rpm and run for 3, 6, 9, 12, 15, 18, 21, or 30 s (*n* = 5 for each duration) using either a 4.0-mm diamond burr or a 4.0-mm steel burr. Immediately after drilling a bovine femur, the temperature of the burr tip was measured using a calibrated thermometer (FG-100B Tip Thermometer, Hakko Corporation, Osaka, Japan). In addition, to evaluate how much heat passed through the bone tissue from the device tip, an additional experiment was performed. The thoracic lamina bone of mice was first thinned using a high-speed drill to simulate the actual surgical procedure, and then the thinned bone was harvested from the mice. The harvested lamina was placed between the tip of the digital soldering station and a thermometer. The temperature reaching the sensor was recorded using tip settings of 120 °C, 90 °C, and an unheated control (room temperature).

### Thermal-induced spinal cord injury model

Mice were deeply anesthetized with isoflurane (4% for induction and 2% for maintenance). A 20-mm midline skin incision was made over the spine, and a laminectomy of the T8 laminae was performed to expose the spinal cord. The exposed spinal cord at the T10 spinal segment was then subjected to a thermal injury (90 °C for 1 min) using a digital soldering station (FX-888D, Hakko Corporation). To confirm the actual temperature applied to the dura mater, the tip temperature was measured with a precision thermometer immediately before and after contact with the spinal cord. In the thermal injury model, the tip was maintained at a constant 90 °C for the entire 1-min duration (Supplementary Figure 1A). The tip of the device was gently placed in direct contact with the dorsal surface of the dura mater (Supplementary Figures 2A,B). After the injury, the overlying muscles were sutured in layers. During recovery from anesthesia, the animals were placed in a temperature-controlled chamber until they regained normal thermoregulation. To confirm that the injury was caused only by the thermal insult, a sham-operated group was established during the model development phase. These mice underwent laminectomy and placement of the unheated device tip on the dorsal dura mater for 1 min. In the sham operation, the tip temperature was confirmed to be at room temperature (approximately 22–26 °C) both before and after the 1-min contact (Supplementary Figure 1A). A naive (unoperated) group was also included as an intact control. Since the behavioral assessment confirmed that there were no functional differences between the sham-operated and naive mice (see Results), naive mice were utilized as the primary control for the subsequent histological and transcriptomic analyses.

### Locomotor function analysis

The ladder rung walking task (LRWT) was performed to evaluate skilled walking and paw placement (Metz and Whishaw, 2009). The apparatus consisted of a horizontal ladder (100 cm long) with transparent acrylic walls (20 cm high) and metal rungs (0.2 cm diameter) spaced 1 cm apart. The ladder was elevated 30 cm above the ground. Mice were placed at one end of the ladder and allowed to walk across to reach their home cage at the opposite end. Each trial was recorded from a lateral view using an iPad Air camera (5th generation, Apple Inc., Cupertino, CA, United States) at a frame rate of 60 frames per second (fps). Forelimb and hindlimb placement was assessed using a quantitative foot fault scoring system. The skilled walking performance score (SWPS) was calculated as follows: SWPS (%) = (Total score/(Number of cycles × 6)) × 100 (Martins et al., 2022). A footprint analysis was performed as described previously (Kitade et al., 2023). Briefly, the mice were then allowed to walk along a paper-covered runway (4 cm wide × 80 cm long) placed between a brightly lit starting box and a dark goal box containing a food reward. The forepaws and hindpaws were dipped in red and green dyes, respectively.

### Quantitative real-time polymerase chain reaction

The mice were euthanized by cervical dislocation under deep isoflurane anesthesia (4% for induction and 2% for maintenance). Total RNA was isolated from each injured spinal cord specimen (a 4-mm segment centered on the lesion epicenter) using an RNeasy Mini Kit (Qiagen, Hilden, Germany). The RNA was reverse transcribed using PrimeScript Reverse Transcriptase (TaKaRa, Shiga, Japan). Quantitative real-time PCR was performed on a 20 μL reaction mixture containing primers specific to the genes of interest (Supplementary Table 1) and TB Green Premix DimerEraser (TaKaRa). The mRNA levels in each sample were normalized to that of glyceraldehyde-3-phosphate dehydrogenase (GAPDH). Real-time PCR was conducted using a CFX Connect Real-Time PCR Detection System (Bio-Rad, Hercules, CA).

### Bulk RNA barcoding and sequencing

Spinal cord specimens were collected using the same procedure as described for quantitative real-time polymerase chain reaction (qRT-PCR). Libraries were prepared for bulk RNA barcoding and sequencing (BRB-seq) with some modifications to the original protocol (Alpern et al., 2019). An oligo(dT)-based primer was used for first-strand cDNA synthesis, and the Second Strand Synthesis Module (NEB, #E6111) was used for second-strand synthesis. In-house MEDS-B Tn5 transposase was used for tagmentation (Picelli et al., 2014; Sato et al., 2019). The resulting fragments were amplified by 10 cycles of PCR using Phusion High-Fidelity DNA Polymerase (Thermo Scientific, #M0530). Sequencing was performed on an Illumina NovaSeq 6000 platform to obtain 81-bp insert reads (Read 2).

### BRB-seq data processing

Barcodes (Read 1) were extracted using UMI-tools (v1.1.1) with the following command: umi_tools extract -I read1.fastq --read2-in = read2.fastq --bc-pattern = NNNNNNNNNNCCCCCCCCC --read2-stdout. Adaptor sequences and low-quality bases were trimmed, and reads shorter than 20 bp were discarded using Trim Galore! (v0.6.10). Reads were then mapped to the GRCm38 mouse reference genome using HISAT2 (v2.2.1). Read counts per gene for each sample were generated from the resulting BAM files using featureCounts (v2.0.6). Differentially expressed genes (DEGs) were identified using DESeq2 (v1.40.2) with thresholds of an absolute log2 fold change >1 and an adjusted *p*-value (*p*-adj) < 0.05.

### Histopathological examination

Mice were deeply anesthetized with isoflurane (4% for induction and 2% for maintenance). After thoracotomy, a 23-gauge winged needle was inserted into the left ventricle, and transcardial perfusion with ice-cold normal saline was performed. The right atrium was incised to allow exsanguination. After euthanasia was confirmed by transcardial saline perfusion, mice were transcardially perfused with 4% paraformaldehyde (PFA). The spinal cords were removed, post-fixed in 4% PFA for 24 h, and then cryoprotected by sequential immersion in 10% sucrose for 1 h and 30% sucrose for 24 h. The spinal cords were embedded in optimal cutting temperature (OCT) compound. Frozen sections were cut at 16 μm (sagittal) or 10 μm (axial) using a cryostat. Sections were incubated with primary antibodies overnight at 4 °C, followed by a 1-h incubation with the corresponding fluorophore-conjugated secondary antibodies and Hoechst 33342 (1:1,000; Invitrogen). The primary antibodies used were as follows: anti-Myelin Basic Protein (MBP, rat, 1:400; Millipore) and anti-5-hydroxytryptamine (5-HT, goat, 1:400; ImmunoStar). The area of spared white matter, the area of collagen deposition, the number of Col1a2-EGFP^+^ cells/mm^2^, the rostrocaudal length of the Col1a2-EGFP^+^ and MBP^−^ regions, and the 5-HT^+^ area were evaluated in one section from the lesion epicenter per animal (*n* = 6 mice per group).

### Image acquisition and quantitative analysis

All images were obtained using a BZ-X710 digital microscope system (Keyence, Osaka, Japan). The area of spared white matter was quantified on transverse sections stained with Luxol Fast Blue (LFB) (*n* = 6 mice per group), as previously described (Saiwai et al., 2010). Similarly, the area of collagen deposition was quantified on transverse sections stained with Picrosirius Red (*n* = 6 mice per group), as previously described (Yokota et al., 2017). The number of Col1a2^+^ cells/mm^2^ was counted using Dynamic Cell Count software (BX-H1C, Keyence) (Saito et al., 2017). The rostrocaudal lengths of the GFP^+^ and MBP^−^ regions, and the 5-HT^+^ area were measured using ImageJ (National Institutes of Health, Bethesda, MD, United States).

### Statistical analysis

All statistical analyses were performed using GraphPad Prism (version 10.5.0; GraphPad Software Inc., San Diego, CA). Data are presented as mean ± SEM. A *p*-value <0.05 was considered statistically significant. Footprint analysis and LFB staining results were compared using *t*-test. Results from the LRWT, Picrosirius Red staining, Col1a2^+^ cell counts, GFP^+^ and MBP^−^ region lengths, and 5-HT^+^ area counts were analyzed by a one-way factorial analysis of variance (ANOVA) with a *post-hoc* Tukey–Kramer test.

## Results

### The drill tip reached high temperatures during bone drilling

**Figure 1:**
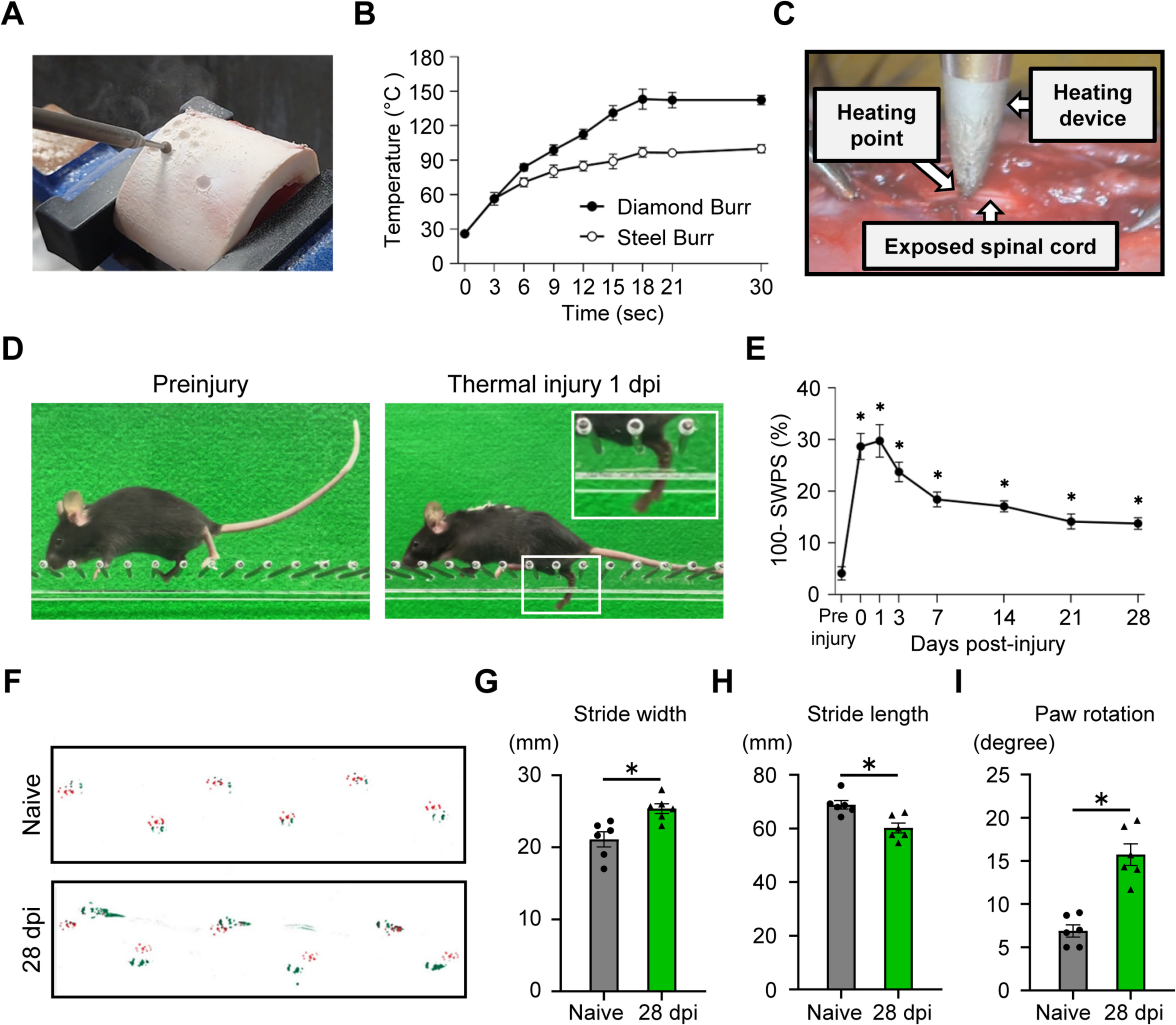
Establishment and functional characterization of a mouse model of thermal-induced spinal cord injury (TiSCI). **(A)** Experimental setup for measuring burr tip temperature during bone drilling with a high-speed drill. **(B)** The time course of burr tip temperature during bone drilling with a diamond or a steel burr. The tip temperature of both burrs reached 90 °C within 30 s (*n* = 5 per time point). **(C)** Schematic illustration of the surgical procedure for the mouse model of TiSCI. A temperature-controlled heating device (90 °C for 1 min) was applied to the exposed thoracic spinal cord. **(D)** Representative photographs of mice performing the ladder rung walking task before TiSCI (naive) and at 1 day after TiSCI. The injured mouse exhibits prominent hindlimb foot faults. **(E)** The time course of the skilled walking performance score (SWPS) for 28 days after TiSCI. Motor deficits persisted throughout the 4-week period (*n* = 12 per group). **(F–I)** Footprint analysis at 28 days after TiSCI. The forepaws and hindpaws were dipped in red and green dyes, respectively. **(F)** Representative footprints from a naive mouse and a mouse after TiSCI. Quantitative analysis of hindlimb **(G)** stride width, **(H)** stride length, and **(I)** paw rotation. Mice after TiSCI showed significant locomotor impairment compared to naive mice in all parameters (*n* = 6 per group).

To determine the extent of temperature increase at the drill tip during bone drilling, we used a surgical high-speed drill to measure the tip temperature while drilling a bovine femur with a diamond or steel burr (Figure 1A). With the diamond burr, the tip temperature rose to approximately 90 °C within 6 s of drilling. The tip temperature of both the diamond and steel burrs exceeded 90 °C at 30 s of drilling (Figure 1B). These results suggest that the spinal cord could be susceptible to thermal damage from the heat generated during surgical drilling procedures. We quantified the thermal dynamics of our model approach by measuring the heat that passed through the bone. When mouse lamina bone was placed between the device tip and the sensor, we confirmed that substantial thermal energy reached the spinal cord side. Specifically, with the tip set to 120 °C, the temperature reached 88.83 ± 5.19 °C (mean ± SD), and at a 90 °C setting, it reached 77.67 ± 1.86 °C (Supplementary Figure 3A). When using a non-heated tip, the temperature remained at room temperature regardless of whether the lamina bone was placed between the device tip and the thermometer (Supplementary Figure 3B). Because anatomical variations and the residual bone thickness during drilling differ among individuals, measuring heat transfer through the bone can make the final temperature reaching the spinal cord difficult to predict. To ensure high reproducibility and a consistent thermal dose for every animal, we decided to remove the bone and apply the heating tip directly to the dura mater. Since surgical drilling with diamond burrs can easily exceed 120 °C, these results show that approximately 90 °C of heat can reach the spinal cord side through the bone. Based on these findings, we utilized the 90 °C direct-contact setting as a standardized and effective dose to represent the actual thermal dose received clinically and to clearly characterize the resulting pathological changes.

### Establishment of a novel mouse model of thermal-induced spinal cord injury

To investigate the pathophysiology of TiSCI, we developed a mouse model in which a quantifiable thermal stimulus induces neurological deficits. A heating device capable of precise temperature regulation was applied directly to the exposed thoracic spinal cord under a microscope (Figure 1C). Following TiSCI, we did not observe any physical burning or structural destruction of the tissue under the surgical microscope. When the soldering tip was removed from the spinal cord, no tissue adhered to the device tip, and we confirmed that there was no physical damage to the dura mater or the underlying spinal cord (Supplementary Figures 4A,B). Application of a 90 °C thermal injury to the spinal cord for 1 min induced significant hindlimb motor deficits (Figure 1D). The ladder rung walking task revealed motor deficits immediately after the injury, which persisted for up to 28 days post-injury (dpi) (Figure 1E). Similarly, footprint analysis confirmed that hindlimb motor dysfunction remained at 28 dpi (Figures 1F–I). To ensure that the neurological deficits were caused by thermal damage and not by the surgical procedure itself, we validated the model approach by comparing sham-operated mice (laminectomy with unheated device contact) and naive mice. The SWPS analysis revealed no significant differences between the two groups (Supplementary Figures 5A,B), confirming that the laminectomy and mechanical contact alone do not induce functional impairment. Based on these results confirming the functional equivalence of the two groups, naive mice were selected as the primary control for subsequent analyses to represent the intact state.

### Upregulation of fibrosis-related genes in the spinal cord after TiSCI

**Figure 2:**
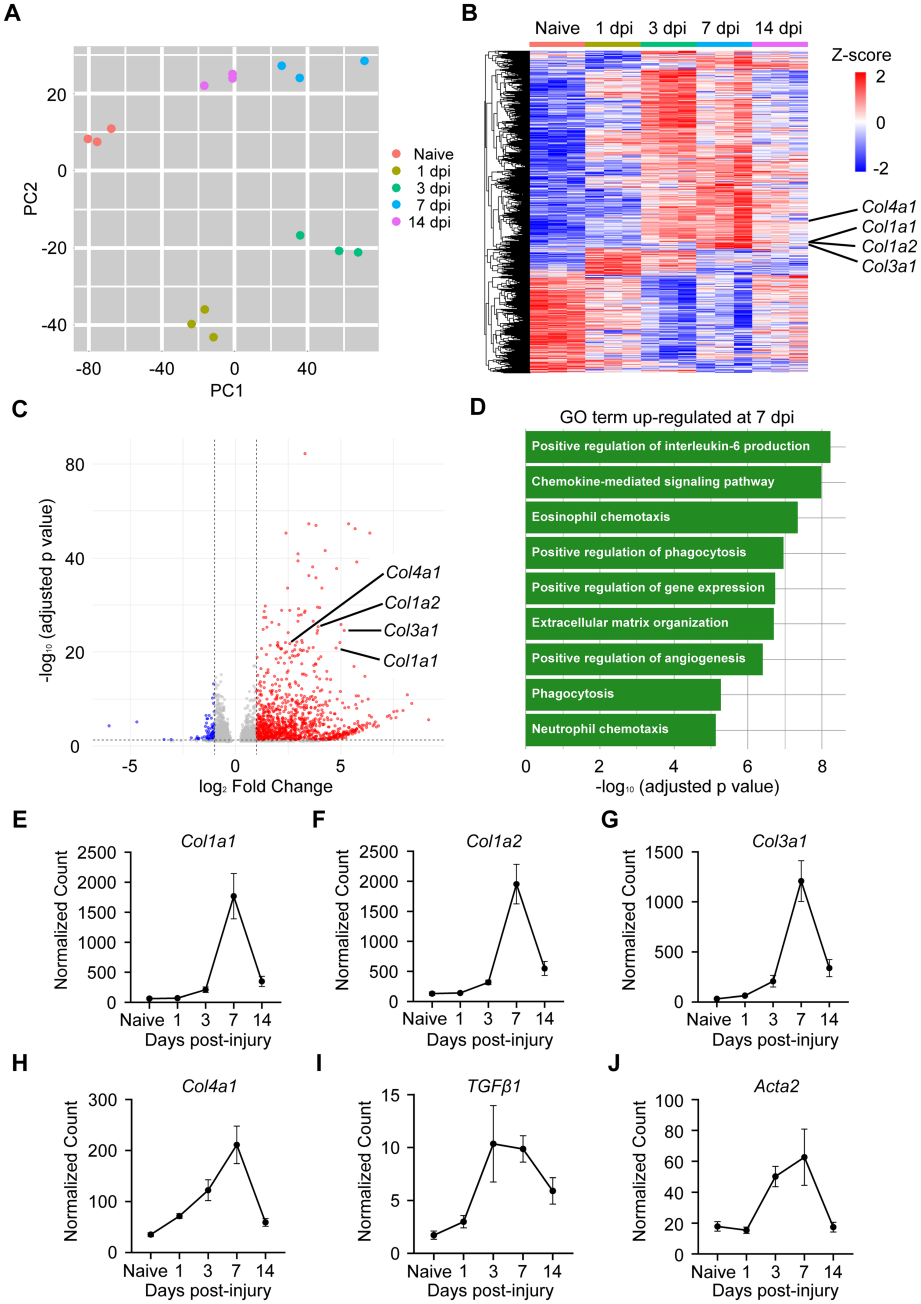
Transcriptomic analysis reveals upregulation of fibrosis-related genes in the thermally injured spinal cord. **(A)** Principal component analysis (PCA) plot based on the expression of all detected genes from naive spinal cords and injured spinal cords at 1, 3, 7, and 14 days after TiSCI. Each point represents a single sample (*n* = 3 per group), and samples from each time point form distinct clusters. **(B)** Heatmap displaying the expression profiles of all detected genes across all time points. This global view visualizes the dynamic temporal changes in gene expression following injury. Each row represents a single biological replicate. Upregulated genes are shown in red and downregulated genes are in blue. **(C)** Volcano plot of differentially expressed genes (DEGs) at 7 dpi compared to the naive group. Red dots represent significantly upregulated genes, and blue dots represent significantly downregulated genes. Key collagen genes (Col1a1, Col1a2, Col3a1, and Col4a1) are highlighted. **(D)** Gene Ontology (GO) analysis of upregulated DEGs at 7 dpi, showing the significantly enriched biological process terms, including extracellular matrix organization. **(E–J)** Time course of normalized counts from RNA-seq for key pro-fibrotic genes: **(E)** Col1a1, **(F)** Col1a2, **(G)** Col3a1, **(H)** Col4a1, **(I)** Tgfβ1, and **(J)** Acta2 (*n* = 3 per group). Data are presented as mean ± SEM.

To elucidate the molecular mechanisms underlying the persistent locomotor deficits observed in our TiSCI model, we performed a transcriptome-wide analysis using RNA-sequencing (RNA-seq) on spinal cord tissue from the lesion epicenter. Principal component analysis (PCA) showed distinct gene expression profiles for naive mice and mice at 1, 3, 7, and 14 dpi, indicating significant temporal changes. Furthermore, the tight clustering of biological replicates (*n* = 3 per group) within each time point demonstrates the high reproducibility of our experimental data (Figure 2A). A heatmap also illustrated these time-dependent differences, notably revealing an upregulation of collagen genes, including Col1a1, Col1a2, Col3a1, and Col4a1, after TiSCI (Figure 2B). A volcano plot comparing gene expression between naive samples and samples at 7 dpi confirmed the significant upregulation of numerous genes, including these collagen genes (Figure 2C). Gene Ontology (GO) analysis of DEGs revealed a significant enrichment of terms related to fibrosis and fibrotic scar formation, such as extracellular matrix organization and positive regulation of angiogenesis (Figure 2D). The time-course of gene expression changes was confirmed by normalized counts from RNA-seq, which revealed the upregulation of Col1a1, Col1a2, Col3a1, and Col4a1 at 7 dpi (Figures 2E–H). We also observed an upregulation of Tgfβ1, a pro-fibrotic factor, at 3 dpi (Figure 2I), followed by an increase in Acta2 (α-SMA), a myofibroblast marker, at 7 dpi (Figure 2J). Consistent with the RNA-seq data, qRT-PCR analysis confirmed that the expression of these collagen genes was significantly upregulated at 7 dpi compared to naive controls (Supplementary Figure 6A). Taken together, these results demonstrate that multiple genes promoting fibrosis were significantly upregulated in the spinal cord following TiSCI.

### Pathological evaluation of the spinal cord after TiSCI

**Figure 3:**
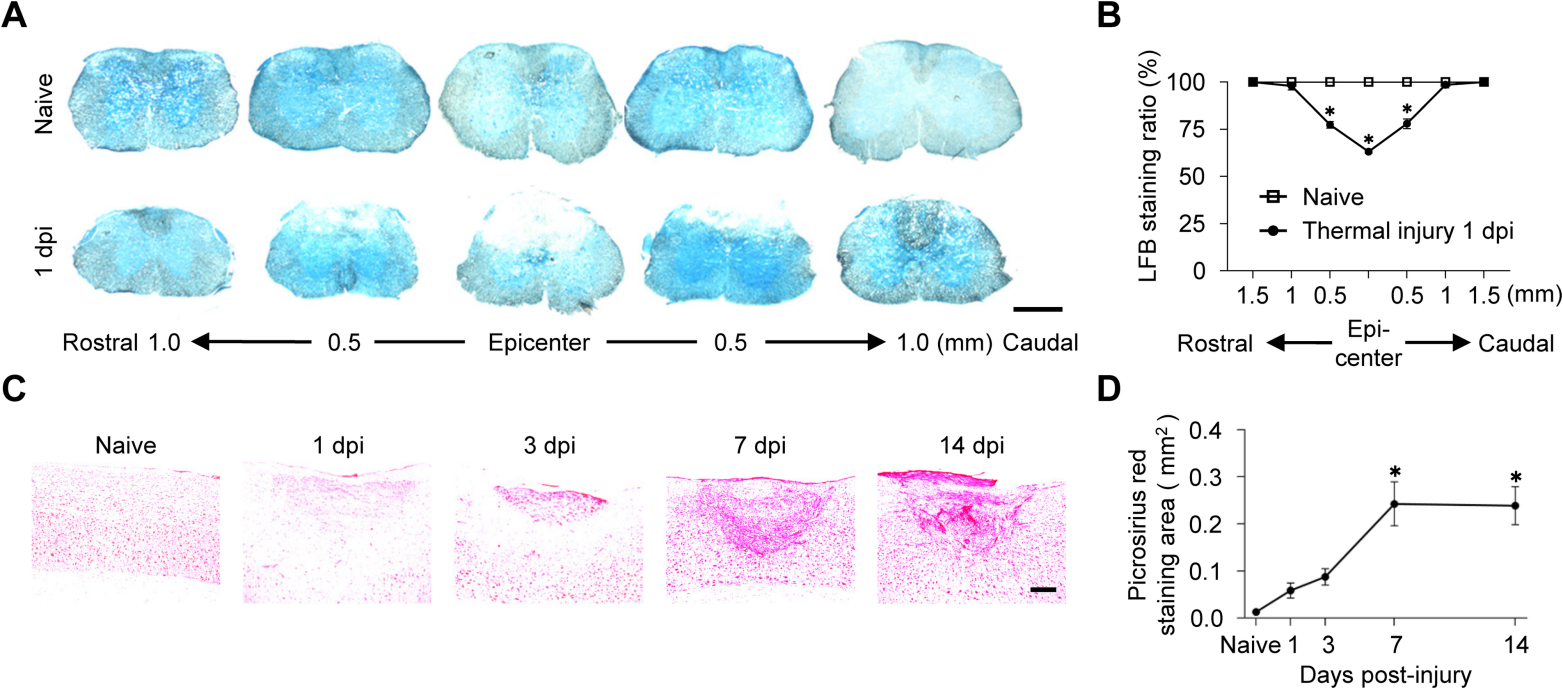
Histopathological analysis of myelin loss and collagen deposition in the thermally injured spinal cord. **(A)** Representative images of Luxol Fast Blue (LFB) staining for myelin in axial spinal cord sections from naive mice and at 1 day after TiSCI. **(B)** Quantification of the LFB staining ratio in axial sections (*n* = 6 per group). **(C)** Representative images of Picrosirius Red staining for collagen in sagittal spinal cord sections from naive mice and at 1, 3, 7, and 14 days after TiSCI. **(D)** Quantification of the PSR-positive area over time (*n* = 6 per group). Data are presented as mean ± SEM. Scale bars: **(A)** 500 µm; (C) 200 µm.

To investigate the histopathological basis for the persistent motor deficits and the pro-fibrotic gene signature identified by our transcriptomic analysis, we next conducted a detailed histological evaluation of the spinal cord after TiSCI. We performed Luxol Fast Blue (LFB) and Picrosirius Red (PSR) staining. LFB staining revealed demyelination extending rostrally and caudally from the lesion epicenter (Figures 3A,B). PSR staining confirmed collagen deposition at the demyelinated injury site (Figure 3C). The PSR-positive area peaked at 7 dpi and persisted until 14 dpi (Figure 3D).

### Fibrotic scar formation and axonal disruption in the spinal cord after TiSCI

**Figure 4:**
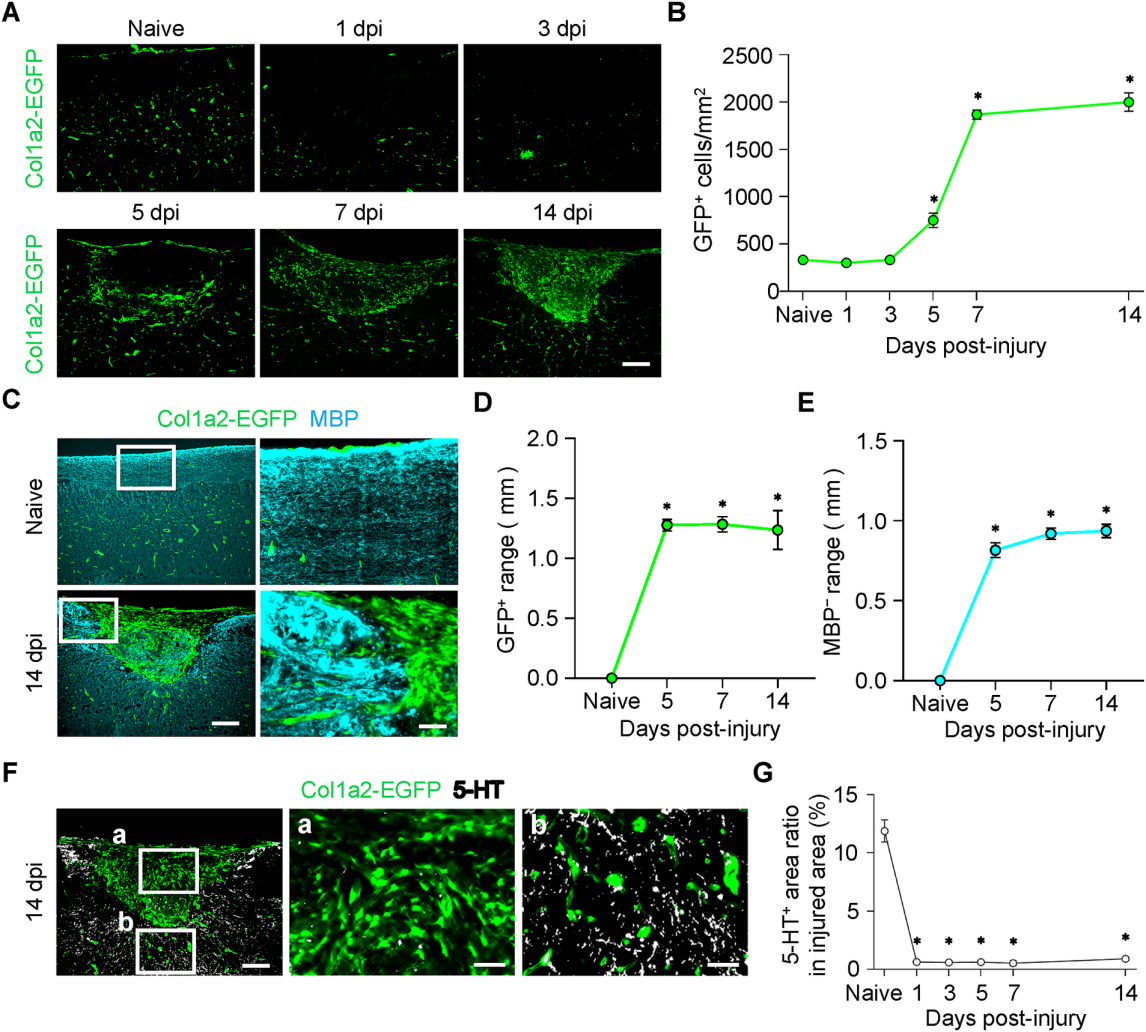
Fibrotic scar formation spatially overlaps with demyelination and axonal disruption after thermal-induced spinal cord injury (TiSCI). **(A)** Representative images of sagittal spinal cord sections from Col1a2-EGFP mice at 1, 3, 5, 7, and 14 days after TiSCI, showing Col1a2-EGFP^+^ cells (green). **(B)** Quantification of the number of Col1a2-EGFP^+^ cells at the lesion epicenter over time (*n* = 6 per group). **(C)** Immunofluorescence staining for Col1a2-EGFP (green) and myelin basic protein (MBP, cyan) at 14 days after TiSCI. The area of Col1a2-EGFP^+^ cell accumulation spatially overlaps with the MBP-negative demyelinated region. **(D,E)** Quantification of the rostrocaudal length of the Col1a2-EGFP^+^ area **(D)** and the MBP-negative area **(E)** over time (*n* = 6 per group). Both regions show a similar temporal distribution. **(F)** Immunofluorescence staining for Col1a2-EGFP (green) and serotonergic (5-HT, white) axons at 14 days after TiSCI. High-magnification insets show that an area with sparse Col1a2-EGFP^+^ cells contains abundant 5-HT^+^ axons (b). In contrast, the prominent Col1a2-EGFP^+^ scar is characterized by a marked reduction of 5-HT^+^ axons (a). **(G)** Quantification of the number of 5-HT^+^ axon profiles at the lesion epicenter over time (*n* = 6 per group). Data are presented as mean ± SEM. Scale bars: 200 µm (**A**,**C** left, **F** left); 50 µm (**C** right, **F** middle and right).

We further used Col1a2-EGFP transgenic mice to histologically characterize the temporal and spatial dynamics of collagen-producing cells during scar formation. Immunostaining revealed the formation of a Col1a2-EGFP-positive fibrotic scar, which progressed from the lesion border toward the epicenter, starting from 5 dpi (Figure 4A). The density of GFP^+^ cells increased, peaking at 7 dpi and remaining significantly elevated until 14 dpi (Figure 4B). The area of the fibrotic scar spatially coincided with the MBP-negative region (Figures 4C–E). Furthermore, we quantified descending serotonergic axons by immunostaining for 5-HT. The 5-HT-positive area in the lesion epicenter was significantly reduced from 1 dpi and remained low until 14 dpi (Figures 4F,G). The observation that axons did not regenerate into the fibrotic scar, which formed from 5 dpi and persisted until 14 dpi (Figures 4A,F,G), suggests that the fibrotic scar may act as an inhibitor of axonal regeneration.

### Activation of pathways promoting fibrotic scar formation after TiSCI

**Figure 5:**
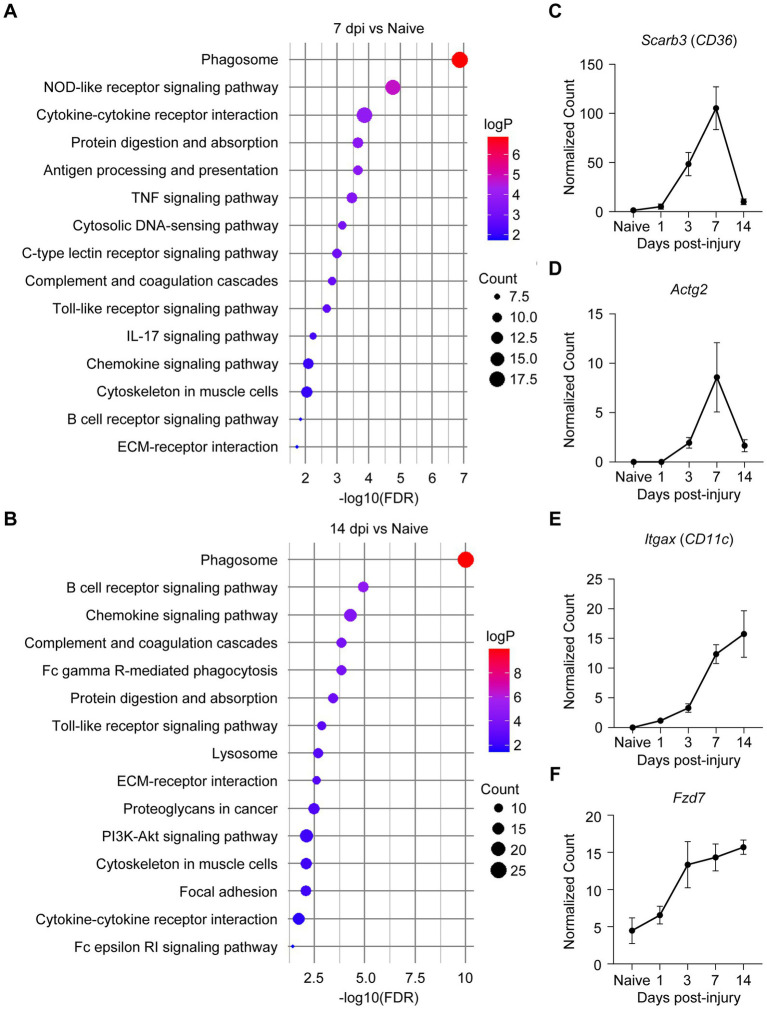
KEGG pathway analysis identifies key driver genes and their associated signaling pathways in the fibrotic response. **(A)** KEGG pathway enrichment analysis of upregulated DEGs at 7 days after TiSCI. The plot displays top enriched pathways, which are primarily associated with phagocytosis and extracellular matrix organization. The size of each circle corresponds to the number of genes in the pathway, and the color intensity indicates statistical significance (adjusted *p*-value). **(B)** KEGG pathway enrichment analysis of upregulated DEGs at 14 days after TiSCI, revealing enrichment of pathways related to opsonin-mediated phagocytosis and Wnt signaling. The plot is displayed as in **A**. **(C,D)** Time course of normalized expression for key driver genes contributing to the pathway enrichments shown in **A** and peaking at 7 days after TiSCI: the scavenger receptor Scrb3 (CD36) **(C)** and the myofibroblast-associated gene Actg2 **(D)**. **(E,F)** Time course of normalized expression for key driver genes contributing to the pathway enrichments shown in **B** and peaking at 14 days after TiSCI: the antigen-presenting cell marker Itgax (CD11c) **(E)** and the Wnt/β-catenin pathway receptor Fzd7 **(F)**. Data are based on normalized counts from RNA-seq and are presented as mean ± SEM (*n* = 3 per group).

Given that our transcriptomic and histological data strongly implicated fibrosis as a key driver of pathology in TiSCI, we next performed a Kyoto Encyclopedia of Genes and Genomes (KEGG) pathway analysis to identify the specific biological pathways underlying this robust fibrotic response. At 7 and 14 dpi, pathways contributing to extracellular matrix (ECM) production and remodeling, such as ECM-receptor interaction, cytokine–cytokine receptor interaction, and Protein digestion and absorption, were significantly enriched (Figures 5A,B). Pathways involved in phagocytosis and antigen presentation were also enriched, including NOD-like receptor signaling pathway and Antigen processing and presentation at 7 dpi; Fc gamma R-mediated phagocytosis and Lysosome at 14 dpi; and Phagosome and Toll-like receptor signaling pathway at both time points. Examination of the DEGs within these pathways revealed an upregulation of the scavenger receptor Scrb3 (CD36) (Figure 5C) and Actg2, which is associated with myofibroblast differentiation, at 7 dpi (Figure 5D). At 14 dpi, we observed an upregulation of Itgax (CD11c), associated with macrophage/microglia activation (Figure 5E), and Fzd7, an indicator of the Wnt/β-catenin pathway (Figure 5F). These findings suggest that multiple pathways activated after TiSCI are involved in fibroblast activation and subsequent fibrotic scar formation.

## Discussion

This study elucidated the molecular and cellular consequences of thermal injury to the spinal cord, a potential complication of high-speed drilling during spinal surgery. We initially established that the drilling process can generate intense heat, reaching up to 90 °C. We established a novel experimental model approach to TiSCI and provides the first direct evidence that a severe thermal insult to the spinal cord induces persistent neurological deficits by triggering robust fibrotic scar formation. Transcriptomic analysis of the injured spinal cord revealed significant upregulation of a set of genes associated with fibrotic scarring. This molecular finding was corroborated by histological evidence of demyelination and extensive collagen deposition at the lesion site. Critically, by 2 weeks post-injury, a prominent fibrotic scar consisting of Type I collagen-positive cells had formed, which coincided with the physical disruption of both myelinated tracts and descending axons. These results strongly suggest that the formation of a fibrotic scar following thermal injury acts as a critical barrier to axonal regeneration, thereby underlying the persistent functional impairment.

A key contribution of this work is the establishment of a reproducible experimental model approach to TiSCI, utilizing a protocol for applying a quantifiable thermal dose. The development of such a model is critical, as human SCIs exhibit significant heterogeneity in severity, making it ethically challenging to prospectively evaluate new treatments for iatrogenic thermal damage in a clinical setting. A reproducible animal model is therefore indispensable for the detailed elucidation of pathophysiology and for validating the efficacy of therapeutic interventions. Our model also provides a robust platform for identifying specific biomarkers for postoperative thermal injury. In the current study, we selected a thermal condition of 90 °C for 1 min, mimicking the temperatures recorded at the surgical drill tip. In fact, in high-risk surgery for ossification of the spinal ligaments, which requires continuous drilling for several minutes in a narrow surgical field immediately adjacent to the spinal cord, the potential for iatrogenic thermal damage is a significant clinical concern (Hosono et al., 2009; Yamamoto et al., 2013; Takahashi et al., 2025). A critical issue in spinal surgery is the heat generated by high-speed drills. While our measurements showed that drill tips can reach 90 °C, we also found that bone tissue acts as a thermal buffer. Our experiments demonstrated that heat passing through the bone results in a temperature drop. We realize that the study of bone heat conduction is a separate research field; however, these data are vital for clarifying our model’s dynamics. In clinical scenarios, particularly when using diamond burrs for precise bone removal in surgeries for ligament ossification, the bone is often drilled until it is extremely thin. In such high-risk situations, the high heat from the burr tip can reach the spinal cord more directly. Furthermore, a nationwide multicenter prospective study on surgery for thoracic ossification of the posterior longitudinal ligament reported a remarkably high 32.2% incidence of postoperative motor palsy (Imagama et al., 2018). To minimize the risk of thermal injury during spinal surgery, surgeons should employ techniques to control heat generation. For instance, intermittent drilling and the use of constant saline irrigation are established techniques to reduce peak temperature elevation and limit the spread of heat during the drilling process (Kondo et al., 2000). Future investigations should systematically vary the temperature and duration of exposure to characterize the full spectrum of physiological changes, particularly regarding motor and sensory deficits. In addition, from a clinical perspective, differentiating thermal injury from mechanical insults like contusion is crucial for guiding therapeutic strategies. Consequently, identifying biomarkers capable of distinguishing between these distinct injury mechanisms represents a crucial next step for translational research.

To distinguish the effects of heat from the mechanical procedure, we validated that physical contact with the device tip does not cause injury. Monitoring the unheated tip confirmed that it remained at room temperature (22–26 °C) throughout the 1-min contact period, even when the drilled lamina bone was present. This confirms that no friction or heat is generated by the contact itself. Furthermore, behavioral analysis using the ladder rung walking task showed that the sham-operated mice were functionally equivalent to naive mice, with no observed functional deficits. These results demonstrate that the gentle contact of the tip is not a factor in the resulting pathology. We can therefore state that the paralysis in this model is specifically caused by the 90 °C thermal dose rather than the mechanical contact of the device.

The mechanisms underlying cell death in the thermally injured spinal cord are multifaceted (White et al., 2007). Primarily, heat exposure causes direct cellular necrosis through membrane disruption and irreversible protein denaturation. This insult subsequently triggers secondary cell death pathways involving protein degradation and DNA damage (Lukácsi et al., 2024). A particularly interesting phenomenon is the “active thermal bystander effect” (ATBE). In this process, heated cells use intercellular signaling cascades to induce delayed cell death in adjacent, non-heated bystander cells (Purschke et al., 2010). Furthermore, thermal stress is known to elicit excessive production of reactive oxygen species (ROS), oxidative stress responses, and mitochondrial dysfunction (Slimen et al., 2014). Indeed, in contusion SCI models, ROS exacerbate inflammatory cell infiltration and worsen functional outcomes (Kubota et al., 2012). Our transcriptomic analysis revealed a significant upregulation of Scarb3 (CD36), a gene implicated in ROS-generating pathways (Cho et al., 2005), strongly suggesting the critical involvement of secondary injury cascades in this thermal injury model. Therefore, therapeutic strategies targeting not only the immediate thermal insult but also the subsequent secondary mechanisms—including ROS-mediated pathways and ATBE—hold promise for improving the survival of resident neural cells.

Our findings strongly suggest that the formation of a fibrotic scar at the lesion site is a major factor contributing to the observed hindlimb motor dysfunction. Histological analysis revealed a progressive accumulation of Type I collagen-positive cells, leading to the formation of a prominent fibrotic scar. This observation is consistent with the known pathological cascade in fibrotic diseases such as hepatic or pulmonary fibrosis, where interactions among inflammatory and stromal cells, including fibroblasts, promote the differentiation into myofibroblasts and subsequent ECM deposition (Iwaisako et al., 2014; Fang et al., 2025). Our transcriptomic data support this mechanism, showing an upregulation of gene signatures associated with the innate immune responses and an increase in myofibroblasts (Dorrier et al., 2021). The excessive deposition of ECM and resultant tissue stiffening are known to create a physical barrier that impedes axonal regeneration (Martínez et al., 2022). Indeed, our analysis confirmed a clear disruption of axonal tracts within the established scar tissue. Collectively, these molecular and pathological findings lead us to conclude that excessive fibrotic scarring is a key contributor to the persistent motor deficits in our thermal injury model.

KEGG pathway analysis of our RNA-seq data provided significant insights into the molecular pathways driving fibrosis after TiSCI. At 7 and 14 days post-injury, pathways associated with fibroblast activation, ECM production, phagocytosis of necrotic cells and myelin debris, and antigen presentation were significantly upregulated. Specifically, expressed genes peaking at 7 dpi included Scarb3 and Actg2. Scarb3, which encodes a scavenger receptor expressed on macrophages and other cells, is involved in the initial inflammatory response by facilitating the phagocytosis of damage-associated molecular patterns (DAMPs) and cellular debris (Comish et al., 2020; Song et al., 2025). Concurrently, Actg2 promotes the differentiation of fibroblasts into myofibroblasts (Muhl et al., 2020; Li et al., 2022), which drive the fibrotic process by synthesizing and depositing collagen, the primary structural component of the fibrotic scar (Zhang et al., 2020). Subsequently, genes peaking at 14 dpi included Itgax (CD11c) and Fzd7. Itgax is a marker for antigen-presenting cells, such as activated macrophages and dendritic cells, and as a complement receptor, it enhances the clearance of opsonized myelin fragments (Uotila et al., 2013; Borucki et al., 2020; Kohno et al., 2022). Fzd7 is a receptor for the Wnt/β-catenin pathway, which potently drives fibroblast activation, ECM production, and myofibroblast differentiation (von Maltzahn et al., 2011; Guan and Zhou, 2017). This temporal gene expression profile suggests a shift in the phagocytic mechanism at the lesion site, from an early process involving scavenger receptors like Scarb3 to a later, opsonin-dependent clearance mediated by complement receptors like Itgax. We postulate that the persistent presence of DAMPs, such as myelin debris, and the subsequent prolonged phagocytic response may trigger excessive fibrotic scar formation. Therefore, therapeutic strategies aimed at promoting the efficient clearance of myelin debris or modulating fibroblast proliferation and activation could mitigate fibrosis and improve neurological outcomes.

This study has several limitations. First, we did not directly measure the burr tip temperatures during actual human spinal surgeries. In clinical settings where extensive bone drilling is required, such as in patients with severe ossification of the ligamentum flavum (Ricciardi et al., 2021), it is plausible that prolonged surgical times could generate temperatures exceeding the 90 °C used in this study. Second, anatomical variables, such as the thickness of the remaining lamina, may alter the amount of heat transferred to the dura mater and spinal cord, thereby influencing the severity of the injury (Tamai et al., 2017). Third, this system should be viewed as an experimental model approach rather than a traditional disease model. Its primary purpose is to provide a platform for investigating the biological impact of thermal energy on neural tissue and for optimizing surgical conditions. While we utilized a single standardized thermal dose to establish this platform, investigating the dose–response effects of different temperatures and exposure durations remains an important goal for our future research. Finally, a key limitation is that our severe injury paradigm does not perfectly match the actual surgical environment, which includes variables like intermittent drilling and irrigation. However, this controlled approach was a necessary first step to clearly show the pathological potential of thermal insult. Future studies using this platform are needed to explore these variables and to identify non-invasive biomarkers capable of distinguishing between thermal and mechanical injury mechanisms, a crucial step for clinical translation.

Despite these limitations, the animal model established herein offers the distinct advantage of applying a quantifiable and reproducible thermal dose. This study establishes a novel experimental model of TiSCI and provides the first direct evidence that a severe thermal insult to the spinal cord induces persistent neurological deficits by triggering a robust fibrotic scar formation. While direct histological evidence of fibrosis in human iatrogenic SCI is scarce due to ethical limitations on tissue sampling, our findings in a controlled setting highlight a critical, previously unrecognized pathological mechanism. This model serves as an essential preliminary platform for future translational research aimed at optimizing surgical safety and developing therapies targeting fibrosis. This controlled platform allows for detailed characterization of the diverse physiological and pathological resulting from thermal injury. This model is expected to serve as a valuable tool for elucidating the fundamental mechanisms underlying the complex pathologies observed in clinical settings and will provide crucial insights for future translational research. Additionally, our results have significant implications for veterinary medicine, given the frequent use of high-speed drills in animal spinal surgery. The TiSCI model provides essential data for refining surgical protocols and enhancing safety in both human and animal patients, ultimately contributing to the advancement of spinal surgery across species.

## Conclusion

In this study, we established an experimental model approach to TiSCI exhibiting significant neurological deficits. We found that TiSCI led to the upregulation of genes promoting fibrosis and the formation of a fibrotic scar composed of Type I collagen-positive cells. This scar was associated with the disruption of axonal connectivity at the lesion site. These findings suggest that inhibiting the formation of the fibrotic scar may restore the integrity of axonal tracts and promote functional recovery after injury.

## Data Availability

The original contributions presented in the study are publicly available. All sequencing data have been deposited with accession codes PRJDB40340 (PSUB047535) in the DNA Data Bank of Japan (DDBJ). This data can be found here: https://ddbj.nig.ac.jp/search/entry/bioproject/PRJDB40340.
