# Supplementary figures and images for: Establishing an experimental model approach to thermal-induced spinal cord injury in mice
Source: Front Cell Neurosci. 2026 Mar 17;20:1779728. doi: 10.3389/fncel.2026.1779728 (PMC13037712; doi:10.3389/fncel.2026.1779728)

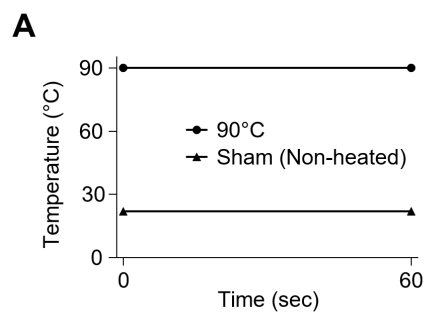

**A**

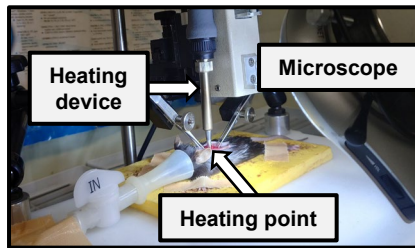

**B**

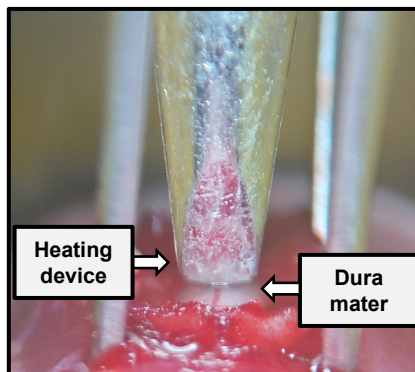

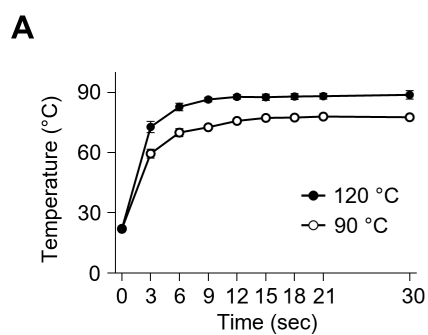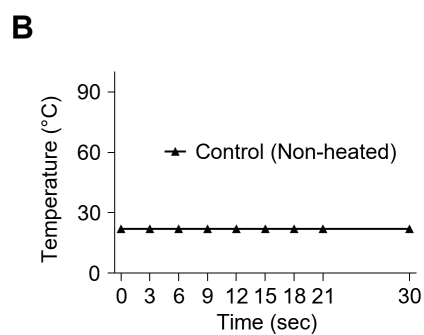

**A**

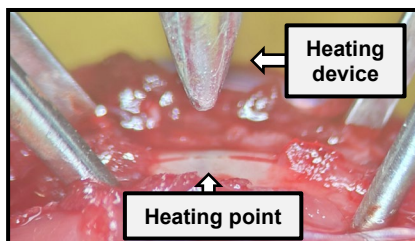

**B**

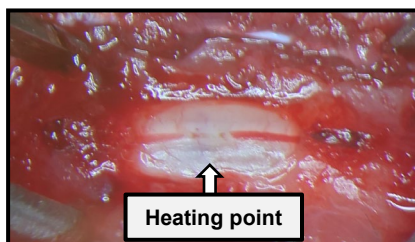

**A**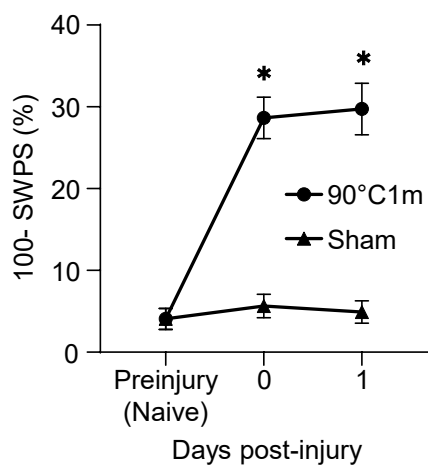**B**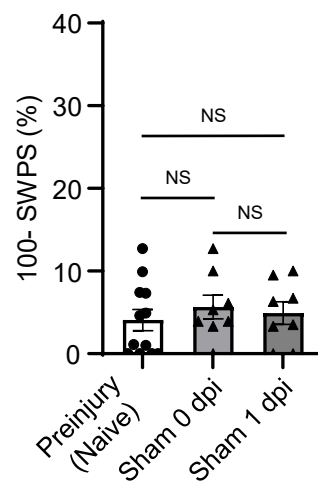

**A**

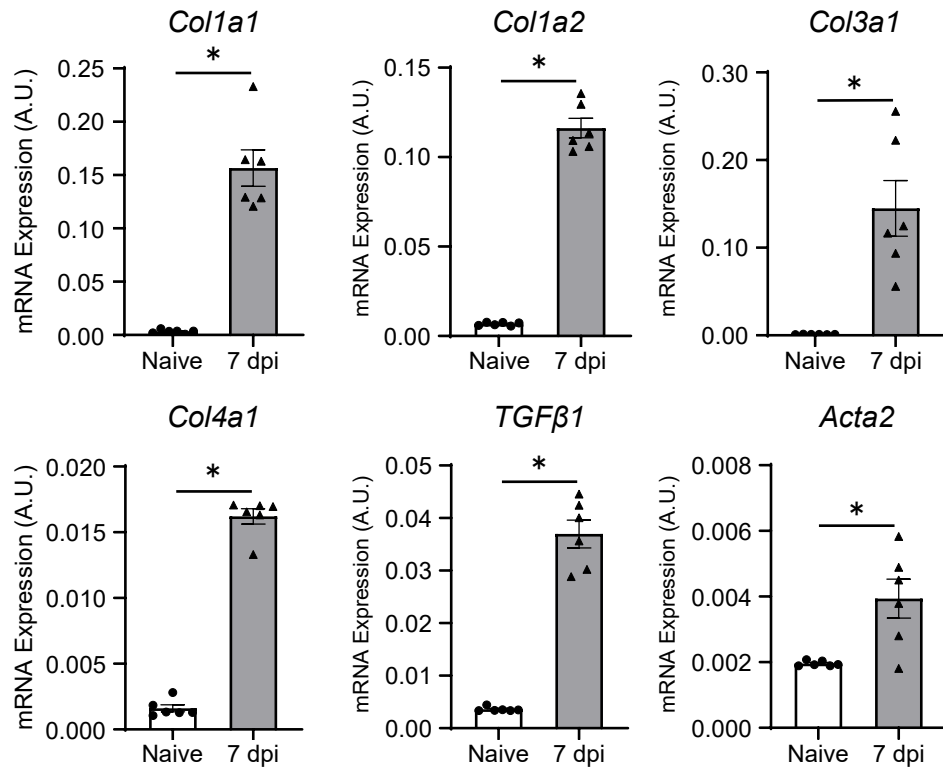

Supplemental figure 6.

Supplement: Supplementary file 1 [file Supplementary_file_1.pdf]
