# Supplementary material for: Establishing an experimental model approach to thermal-induced spinal cord injury in mice
Source: Front Cell Neurosci. 2026 Mar 17;20:1779728. doi: 10.3389/fncel.2026.1779728 (PMC13037712; doi:10.3389/fncel.2026.1779728)
Supplement: Supplementary file 2 [file Table_1.docx]

**Supplemental table 1. Primers used for quantitative RT-PCR**

| Gene symbol | Accession number | 5’- Forward primer -3’ | 5’- Reverse primer -3’ |
| --- | --- | --- | --- |
| *Col1a1* | NM_007742.4 | cctcagaagaactggtacatcagc | ctacgctgttcttgcagtgatagg |
| *Col1a2* | NM_007743.3 | agtcgatggctgctccaaaa | atttgaaacagacggggcca |
| *Col3a1* | NM_009930.2 | taaagaagtctctgaagctgatgg | atctatgatgggtagtctcattgc |
| *Col4a1* | NM_009931.2 | caattaggcaggtcaagttctagc | tggctatctatacacctcctctgg |
| *Tgfb1* | NM_011577.2 | gctgaaccaaggagacggaa | gaagggccggttcatgtca |
| *Acta2* | NM_007392.3 | tccagccatctttcattgggat | ctgtcagcaatgcctgggta |
| *Gapdh* | NM_001289726.2 | atgaatacggctacagcaacaggg | gtctgggatggaaattgtgaggga |
